# Overexpression of *miR-17* is correlated with liver metastasis in colorectal cancer

**DOI:** 10.1097/MD.0000000000019265

**Published:** 2020-02-28

**Authors:** Hao Lai, Jie Zhang, Hongqun Zuo, Haizhou Liu, Jing Xu, Yan Feng, Yuan Lin, Xianwei Mo

**Affiliations:** a Gastrointestinal Surgery Department, Guangxi Medical University Cancer Hospital; b Guangxi Clinical Research Center for Colorectal Cancer; c Research Department, Guangxi Medical University Cancer Hospital, Nanning, Guangxi Zhuang Autonomous Region, P.R. China.

**Keywords:** colorectal cancer, liver metastases, miRNA, overexpression

## Abstract

**Background:**

Colorectal cancer (CRC) is the second leading cause of cancer-related deaths in men and women. The presence of systemic disease, with metastatic spread to distant sites such as the liver, considerably reduces the survival rate in CRC. Cancer stem cells contribute to the metastatic potential of CRC. However, the mechanism underlying metastasis in CRC remains unclear. Thus, this study aimed to examine the expression of microRNAs (miRNAs) in CRC stem cells in cases of liver metastases and assess their correlation with clinicopathological features.

**Methods:**

miRNAs showing high expression in liver metastases and primary lesions were selected through data mining of gene expression omnibus datasets, and miRNAs characteristic of stem cells were selected through COREMINE medical text mining. Subsequently, paired formalin-fixed paraffin-embedded tissue samples of primary CRC and liver metastasis from 30 patients were examined for the expression of miRNAs common to these lists (*hsa-miR-20a, hsa-miR-26b*, *hsa-miR-146a*, *hsa-miR-17*, *hsa-miR-451*, *hsa-miR-23a*, and *hsa-miR-29a*) using quantitative real-time polymerase chain reaction. Further, miRNA expression was compared between liver metastases and the primary tumor in each patient and the factors associated with differential expression were analyzed.

**Results:**

*hsa-miR-17* was significantly upregulated in liver metastases (*P* < .05), but no significant difference in the expression of *hsa-miR-26b*, *hsa-miR-146a*, *hsa-miR-451*, *hsa-miR-23a*, and *hsa-miR-29a* was observed between primary tumors and liver metastases. The higher expression of *hsa-miR-17* in liver metastases was associated with the administration of neoadjuvant chemotherapy and tumor differentiation (*P* < .05) but was not associated with age, sex, tumor location, or lymphatic metastasis.

**Conclusions:**

High expression of *miR-17* may contribute to liver metastasis in CRC. Therefore, an in-depth understanding of its downstream pathways could help in elucidating the mechanisms underlying liver metastases in CRC. However, additional studies are warranted to validate these findings.

## 1 Introduction

Liver metastasis in colorectal cancer (CRC) is a worldwide concern. It is associated with a poor prognosis and reduces the long term survival of patients.^[1]^ There are about 500,000 cases of liver metastasis from CRC annually in USA^[1]^ and until recently, its incidence was still very high.^[2]^ More than 70% of patients with liver metastases cannot undergo surgery. Even among those who undergo hepatectomy, the 2-year recurrence rate is as high as 75%, and the 5-year overall survival rate is only 26.8%. Although metastasis is the main cause of death in the case of such tumors, the complex mechanism underlying metastases in CRC remains poorly understood, and novel and well-characterized biomarkers – which would be helpful in predicting metastatic potential and prognosis of CRC and aid in the facilitation of therapeutic intervention – remain unknown.

MicroRNAs (miRNAs) belong to a class of small noncoding RNAs that can regulate the expression of target genes during the post-transcriptional phase.^[3]^ Growing evidence indicates that miRNAs play an important role in the development of CRC. A high frequency of miRNA dysfunction is associated with CRC development and progression, and miRNAs could thus serve as targets for treatment.^[4–6]^ Thus, miRNAs – many of which have differential expression profiles – may serve as biological markers for predicting the development and prognosis of CRC. Although previous studies have provided valuable data on the potential use of miRNAs as biomarkers in CRC,^[6]^ the results for CRC liver metastasis were limited, and there were inconsistencies in the findings owing to the use of different sample source.^[6,7]^

Cancer stem cells (CSC) was reported to have the ability of self-renewal, sphere formation, migration, invasion, and resistance to cancer therapy such as radiotherapy and chemotherapy and was consider the major contributor of the formation of metastasis. For example, high expression of Oct4 granted cells with ability of cells and associated with the formation of liver metastasis in CRC.^[8]^ Many miRNAs were reported to effect on the stemness of CSC in CRC,^[9]^ including miR-21,^[10]^ miR146a,^[11,12]^ mir-195-5p,^[13]^ and so on.

The aim of the present study was to examine the expression of miRNAs related to CRC stem cells in cases of liver metastases and assess their correlation with clinicopathological features.

## 2 Methods

### 2.1 Data mining of gene expression omnibus (GEO) datasets

In this study, miRNA profiling datasets generated from paired primary tumors and liver metastases of CRC patients were retrieved from the GEO database using the following criteria:

Keywords: colorectal cancer; species: *Homo sapiens*; analysis type: miRNA expression profiling by array.

Data profiles of paired primary tumor and liver metastases were selected manually.

### 2.2 Differentially expressed miRNAs

The online tool GEO2R was used to compare the expression of miRNAs between primary and metastatic lesions. Differentially expressed genes were identified after excluding

(1) miRNAs showing a greater than 1.5-fold increase in expression in less than 20% of samples and
(2) miRNAs for which data were missing in 50% or more of the samples.

After an analysis of the GEO2R results, miRNAs differentially expressed in primary and metastatic lesions were selected. Using overlapping and consistent comparison methods, miRNAs consistently upregulated or downregulated in all chip datasets were selected. Because the difference between the primary and metastatic lesions in this study may be influenced by factors such as smaller stem cell populations, the *P*-value was adjusted to <.05. Moreover, instead of using |logFold Change (FC)|≥2 as the threshold, genes showing consistent differential expression with |logFC|≥1 were defined as the differentially expressed miRNAs.

### 2.3 Text mining of CRC stem cell-related miRNAs

COREMINE-Medical is an ontology-based medical information retrieval platform, jointly developed by the Norwegian and Chinese Academy of Sciences, the Chinese Academy of Medical Sciences, the National Library of Medicine, and other groups and is one of the most advanced medical information retrieval platforms in the world. To identify miRNAs associated with CSC, we used the COREMINE Medical text mining tool and applied it to the Pubmed database. Search keyword combinations included “miRNA,” “Cancer stem cell,” “Colorectal cancer,” “epithelial to mesenchymal transition,” and “Drug Resistance.” On reading through the literature retrieved by COREMINE, several miRNAs were selected as CRC stem cell-related miRNAs.

### 2.4 Tumor specimens

Tissue samples were collected from 30 patients with advanced CRC who were treated in Guangxi Medical University Cancer Hospital (GMUCH) from January 1, 2011 to December 31, 2016. The primary lesion and metastases were removed either simultaneously or at different times using surgery, and the pathology was confirmed by experienced pathologists. All specimens were collected within 1 hour after surgical resection, frozen in liquid nitrogen, and stored in an ultra-low temperature refrigerator at −80°C.

Meanwhile, demographic and clinical information such as age, sex, ethnicity, clinical diagnosis, and pathological type were collected for all patients, who provided signed informed consent before data and sample collection. Moreover, this study was approved by the Ethics Committee of the GMUCH.

### 2.5 RNA extraction

Total RNA was extracted from 50 to 80 mg of tumor tissue using the Beyozol Reagent (Cat. R0011, Beyotime Biotechnology, Shanghai, China) according to the manufacturer's protocol. The purity and concentration of RNA were determined using NanoDrop2000 Spectrophotometers Thermo Fisher Scientific (Waltham, MA, USA) and then stored at −80°C. cDNA was synthesized using the First Strand cDNA Synthesis Kit (TOYOBO, Shanghai Biotechnology Co., Ltd, Shanghai, China) according to the manufacturer's protocol using 1 μg of total RNA.

### 2.6 Real-time polymerase chain reaction (PCR)

Primers for real-time PCR were synthesized by Sangon Biotech (Shanghai, China) Co., Ltd. The primer sequences used are shown in Table 1. miRNA expression was determined using the BeyoFast SYBR Green Qpcr Mix (Beyotime). All experiments were repeated at least thrice, and expression was compared using the −ΔΔCt method.

**Table 1:**
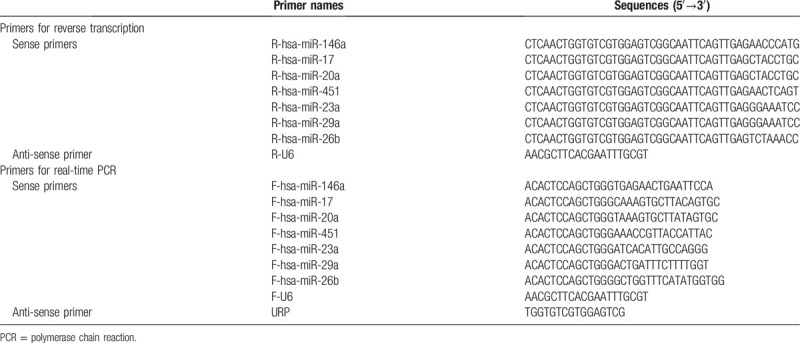
Primers used in reverse transcription and real-time PCR.

### 2.7 Prediction of target genes

TargetScan, miRanda, miRDB, and PicTar were used to identify downstream target genes for miRNAs associated with drug resistance in tumor stem cells, and target genes that could be simultaneously predicted by at least 3 tools were selected for further analysis.

### 2.8 KEGG signal pathway analysis

pathway: Kyoto Encyclopedia of Genes and Genomes (KEGG) pathway analysis was performed using David online analysis tool to explore the possible biological pathways that the predicted target genes of differentially expressed miRNAs were involved in.

### 2.9 Statistical analysis

The data were statistically analyzed using SPSS 13.0 software IBM SPSS Statistics (Armonk, NY, USA). Continuous variables were expressed as mean ± standard deviation (*x* ± *s*), and the differences between groups were analyzed using a paired samples 2-sided *t* test. Discrete variables were expressed as frequency or percentage (%) and were compared using the *χ*^2^ test. Differences with *P* < .05 were considered statistically significant.

## 3 Results

### 3.1 Mining for miRNAs differentially expressed in liver metastasis using GEO

After screening the GEO database for miRNA array analyses involving paired primary CRC lesions and related liver metastases, GSE56350 – completed by an Ohio State University group using the GPL16744 platform and containing data from 33 lymph nodes and 15 pairs of matched primary CRC and liver metastases tissues – was selected for the present study. Using GEO2R analysis, 43 differentially expressed miRNAs were found in both GSE56350 analyses (Table 2), among which 34 genes were upregulated and 9 were downregulated in liver metastases (Table 2).

**Table 2:**
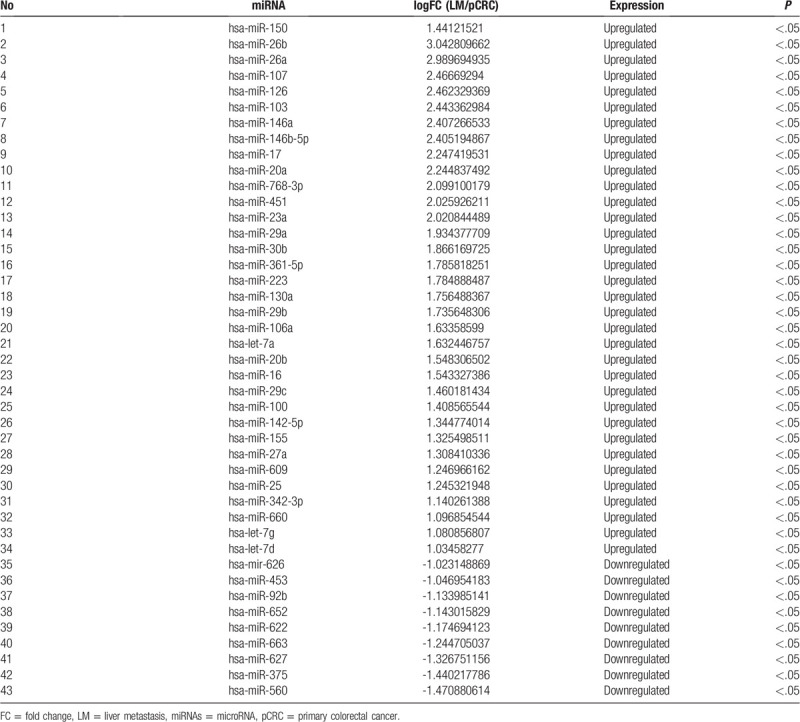
miRNA analysis in pair-match colorectal cancer tissue samples.

### 3.2 Text-mining of CRC stem cell-related miRNAs

Using COREMINE text mining, papers were retrieved using the keywords combinations described in material and methods. After reviewing the literature, 29 related miRNAs, including 11 that were upregulated and 18 that were downregulated, were mined after screening for miRNAs shown to be associated with epithelial mesenchymal transition or self-renewal, asymmetric division, and high drug resistance in CSC (data not shown).

After comparing these miRNAs with those screened by GEO, 7 candidate miRNAs – *hsa-miR-26b*,^[14]^*hsa-miR-146a*, *hsa-miR-17*,^[15]^*hsa-miR-20a*,^[16]^*hsa-miR-451*,^[17]^*hsa-miR-23a*,^[18]^ and *hsa-miR-29a*^[16,19]^ – were selected for further validation (Table 3).

**Table 3:**
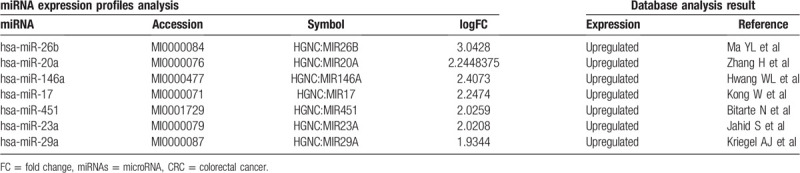
Integrated-signature miRNAs in CRC.

### 3.3 Expression of identified miRNAs in CRC and liver metastasis

The expression of *hsa-miR-26b*, *hsa-miR-146a*, *hsa-miR-17*, *hsa-miR-20a*, *hsa-miR-451*, *hsa-miR-23a*, and *hsa-miR-29a* was examined in 30 liver metastases and paired primary tumor samples from the same patient. Unfortunately, amplification could not be achieved using the primers for *hsa-miR-20a*, *hsa-miR-23a*, *hsa-miR-146a*, and *hsa-miR-451*, and only the upregulation of *hsa-miR-17* in liver metastasis was confirmed (*P* < .05 when compared with the expression in the primary lesion). Although *hsa-miR-29a* also appeared to be differentially expressed, the difference was not statistically significant (*P* *>* .05, data not shown)

### 3.4 Correlation of *miR-17* expression with histopathological features

Patients were classified according to whether they showed higher or lower *miR-17* expression in the liver metastases than in the paired primary colorectal tumors (Table 4). Higher *miR-17* expression in liver metastases was not significantly correlated with sex (*P* = .68), age (*P* = .68), tumor location (*P* = .97), or lymphatic spread (*P* = .19). However, it showed a significant correlation with tumor differentiation (*P* = .03) and the administration of neoadjuvant chemotherapy (*P* = .03).

**Table 4:**
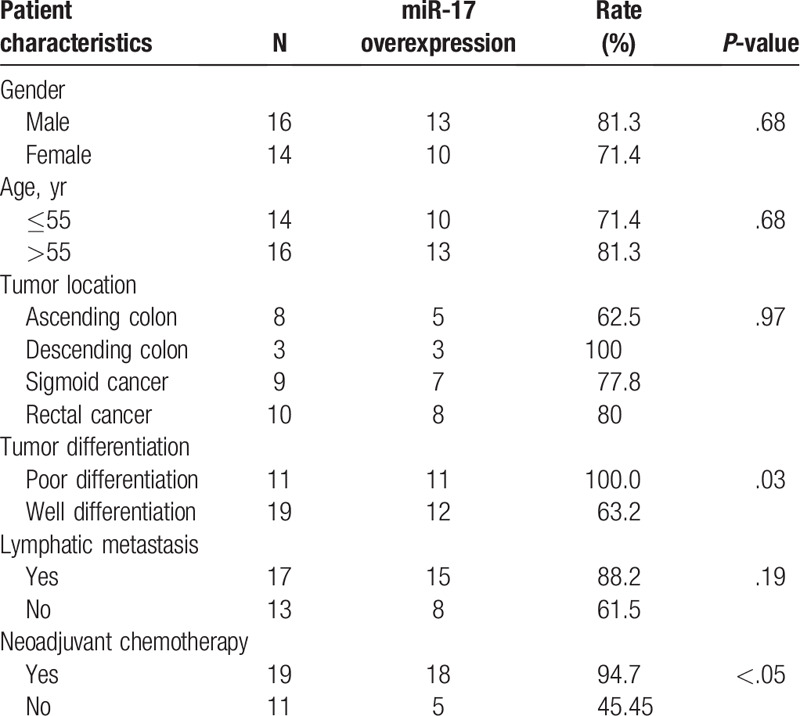
Relationship between the expression level of miR-17 in the primary tumor and clinicopathologic parameters in colorectal cancer patients with liver metastases.

When the patient's primary tumor was poorly differentiated, *miR-17* expression in the metastatic tissue was higher than that in the primary tumor tissue. This trend was true for 100% (11/11) of the poorly differentiated primary tumors, but only for 63.2% of moderately differentiated primary tumors. The expression of *miR-17* was more likely to be higher in liver metastases in patients who received chemotherapy before surgery, whereas only 45.5% of patients who did not receive chemotherapy before surgery showed *miR-17* upregulation in metastatic lesions; this difference was significant (*P* = .03). There was no significant difference in the upregulation of *miR-17* in liver metastases according to sex, age, primary tumor location, or lymph node metastasis (*P* > .05).

### 3.5 Prediction of miR-17 target genes

Through a thorough search of the 4 databases, as mentioned previously, 614 target genes of *hsa-miR-17* were identified and selected for further KEGG pathway. Target genes of *hsa-miR-17* mostly showed enrichment in the endocytosis, MAPK signaling pathway, axon guidance pathway, pathways in cancer, FoxO signaling pathway, etc. The pathways showing a *P*-value < .01 and their key genes were listed in Table 5.

**Table 5:**
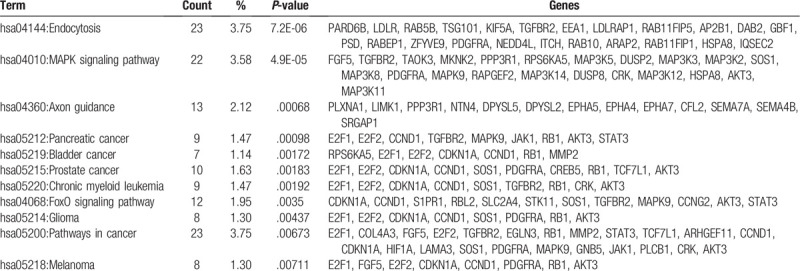
KEGG pathways enrichment of predicted target genes of hsa-mir-17.

## 4 Discussion

CRC is one of the most prevalent malignant tumors worldwide.^[20]^ CSC contribute to the formation of liver metastases^[8,9]^ in such cases, which leads to a poor prognosis.^[21]^ In this study, we identified 7 cancer stem-cell related miRNAs that showed higher expression levels in liver metastases than in primary lesions through a combination of GEO dataset and COREMINE medical text mining. We further validated the expression of those miRNAs in paired primary tumor and liver metastases specimens. Through our study, we confirmed that a higher expression of *hsa-miR-17* in liver metastases was associated with the administration of neoadjuvant chemotherapy and tumor differentiation but not with age, sex, tumor location, or lymphatic metastasis.

*MiR-17* can enhance cell proliferation and metastasis in colon cancer and has been considered a promising biomarker for CRC in several previous studies.^[22]^ Previous reports show that *miR-17* is upregulated in various human cancers, including CRC,^[23–25]^ and the *miR-17* cluster is associated with the progression of colorectal adenoma to adenocarcinoma. Other studies have found that *miR-17* levels continue to rise during the progression of adenoma to adenocarcinoma.^[26]^ Our present study showed that *miR-17* expression increases during progression from the primary tumor to liver metastasis, consistent with findings from Wang et al., who reported a similar *miR-17* expression pattern using qRT-PCR.^[27]^ Our results indicate that *miR-17* may be involved in metastatic spread to the liver in CRC.

However, we were unable to present a large patient cohort for an analysis of *miR-17* expression in primary CRC and the corresponding liver metastases, and larger sample sizes need to be analyzed in order to confirm our findings.

Previous studies revealed some possible mechanisms about how *miR-17* promoted the metastasis. It was shown that *miR-17* could promote CRC cell proliferation and metastasis by targeting transforming growth factor-β receptor 2.^[28]^*MiR-17* could induce drug resistance in CRC cells and negatively regulate PTEN expression.^[29]^ miR-17 was reported to promote hepatocellular carcinoma cells through p38 mitogen-activated protein kinase-heat shock protein 27 pathway.^[30]^ MiR-17 could promote normal ovarian cancer cells to CSC development via suppression of the lkb1-p53-p21/waf1 pathway, but whether it was true in CRC was unknown. In our study, we found that the downstream target genes of *miR-17* were enriched in MAPK pathways, the tyrosine kinase signaling pathway, cell cycle pathways, and insulin signaling pathway (Table 4), all of which are involved in cancer formation. Further studies are necessary to confirm the mechanisms underlying this process.

Although we could not confirm the correlation between the expression of the other 5 CSC-related miRNAs and liver metastases, we cannot exclude the possibility that those miRNAs could also affect metastases of CRC. For example, *miR-23a* is highly expressed in various cancers and acts as an oncogenic miRNA.^[31]^ Previous reports indicate that *miR-23a* functions as a growth-promoting and antiapoptotic factor in hepatocellular carcinoma cells, and it also promotes the growth of gastric adenocarcinoma cells and downregulates the expression of the interleukin-6 receptor.^[32]^ Moreover, *miR-23a* promotes the transition of CRC from the indolent to invasive phenotype and promotes the invasive ability of glioma cells by directly targeting HOXD10.^[33]^ Further, *miR-451* is linked to cancer development and is considered a tumor suppressor based on clinicopathological and cell biological evidence.^[34]^ From a clinicopathological perspective, *miR-451* expression is downregulated in various types of cancers, and its lower expression is correlated with a worse prognosis in cancers such as non-small cell lung cancer, gastric cancer, and hepatocellular cancer.^[35,36]^ As previously reported, *miR-26b* is strongly associated with Ulcerative Colitis-associated Carcinogenesis,^[37]^ and higher expression *miR-26* was reported be associated with metastasis in head and neck squamous cell carcinoma.^[38]^ In prostate cancer, *miR-29a* is considered a putative tumor-suppressive miRNA, contributing to cell migration and invasion.^[39]^ The *miR-29* family plays a dominant role in regulating extracellular matrix genes, such as collagen, LAMA2, integrin β, Mmp2, fibrillin, secreted protein, acidic, and Sparc, consequently contributing to the promotion of cancer cell migration and metastasis. Despite its low expression, we found an upregulation of both *miR-26* and *miR-29* in CRC liver metastases specimens, although this difference was not significant. Thus, the function of these miRNAs in CRC liver metastasis still needs to be elucidated in the future.

In summary, higher *miR-17* expression may contribute to liver metastases of CRC. An in-depth understanding of its downstream pathways could help in elucidating the mechanisms underlying liver metastases in CRC.

## Acknowledgment

The authors would like to thank Editage for English language editing.

## Author contributions

**Data curation:** Jie Zhang, Hongqun Zuo, Yan Feng, Xianwei Mo.

**Formal analysis:** Yan Feng.

**Methodology:** Haizhou Liu, Jing Xu.

**Visualization:** Hao Lai, Haizhou Liu.

**Writing – original draft:** Hao Lai.

**Writing – review and editing:** Hao Lai, Jie Zhang, Yan Feng, Yuan Lin, Xianwei Mo.
